# IgG4- related disease: an orphan disease with many faces

**DOI:** 10.1186/s13023-014-0110-z

**Published:** 2014-07-16

**Authors:** Herwig Pieringer, Ilse Parzer, Adelheid Wöhrer, Petra Reis, Bastian Oppl, Jochen Zwerina

**Affiliations:** 1 Academic Research Unit, 2nd Department of Medicine, General Hospital Linz, Linz, Austria; 2 Paracelsus Medical University Salzburg, Salzburg, Austria; 3 Institute for Pathology and Microbiology, Hanusch-Hospital, Vienna, Austria; 4 Institute of Neurology, Department of Neuropathology, Medical University of Vienna, Vienna, Austria; 5 Department of Internal Medicine 3, University of Erlangen-Nuremberg, Erlangen, Germany; 6 Ludwig Boltzmann Institute of Osteology at the Hanusch Hospital of WGKK and AUVA Trauma Centre Meidling, 1st Medical Department, Hanusch Hospital, Vienna, Austria

**Keywords:** IgG4-RD, Immunoglobulin 4, Storiform fibrosis, Lymphoplasmacytic inflammation

## Abstract

Immunoglobulin G4- related disease (IgG4-RD) is a rare systemic fibro-inflammatory disorder (ORPHA284264). Although patients have been described more than 100 years ago, the systemic nature of this disease has been recognized in the 21^st^ century only. Type 1 autoimmune pancreatitis is the most frequent manifestation of IgG4-RD. However, IgG4-RD can affect any organ such as salivary glands, orbits, retroperitoneum and many others. Recent research enabled a clear clinical and histopathological description of IgG4-RD. Typically, lymphoplasmacellular inflammation, storiform fibrosis and obliterative phlebitis are found in IgG4-RD biopsies and the tissue invading plasma cells largely produce IgG4. Elevated serum IgG4 levels are found in many but not all patients. Consequently, diagnostic criteria for IgG4-RD have been proposed recently. Treatment is largely based on clinical experience and retrospective case series. Glucocorticoids are the mainstay of therapy, although adjunctive immunosuppressive agents are used in relapsing patients. This review summarizes current knowledge on clinical manifestations, pathophysiology and treatment of IgG4-RD.

## Introduction

Immunoglobulin G4- related disease (IgG4-RD) is a systemic fibro-inflammatory disorder of unknown origin. While single organ manifestations have been described already more than 100 years ago, a clear description and nomenclature of this disease have been achieved in the past 10 years. As IgG4-RD may affect virtually every organ, this disease is of interest not only for internal medicine physicians but also for other specialties such as ear nose & throat, dermatology, ophthalmology, and neurology.

### Case vignette

In January 2012, a 61 year- old female presented to a rheumatology unit with a long- standing history of recurrent inflammatory lesions of unknown origin since 1989, which had been previously diagnosed as pseudolymphoma and Sjögren’s syndrome (SS). The leading symptom was bilateral orbital masses, which eventually led to enucleation of the left eye. Further, suspicious enlarged lymph nodes in the head and neck region as well as recurrently enlarged salivary glands were evident. Moreover, paravertebral masses as well as thickening of ocular muscles and nerves were present.

The patient underwent a series of organ biopsies over the years including resections of the orbital pseudotumor and left submandibular salivary gland, as well as biopsies of ocular muscles. All biopsies showed dense lymphoplasmacellular infiltrates of the respective organs. Due to the lack of evidence for monoclonal disease based on these biopsies and inconspicuous bone marrow investigations, the results were interpreted as “pseudolymphoma”. Nonetheless, the patient received treatments such as chlorambucil and radio-chemotherapy. All prednisone- containing treatment schemes were successful in decreasing mass sizes and organ swellings for a short period. However, the patient’s disease course was chronic relapsing during the first years. In the last 8 years before referral, however, the disease changed to a more chronic, stable course without major complications.

**Figure 1:**
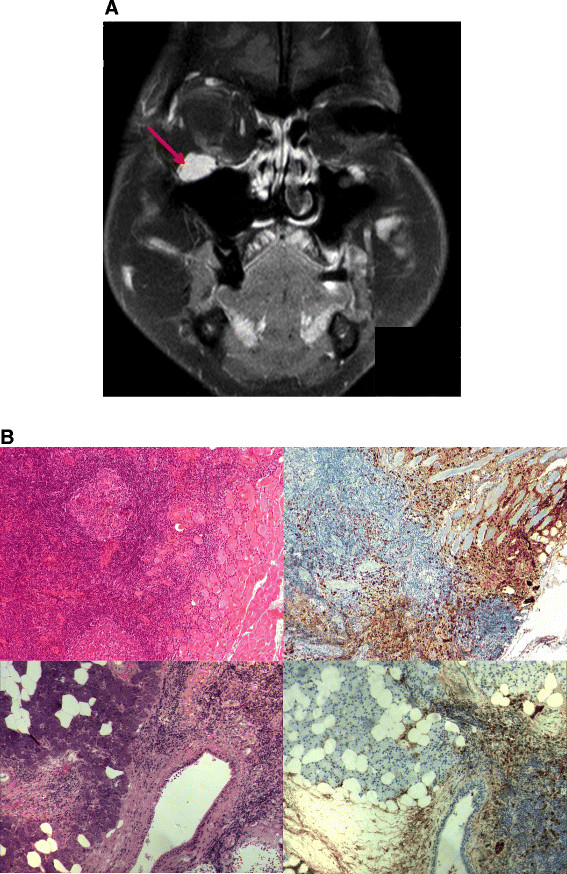
**A: IgG4- related disease with involvement of the orbits, ocular nerves and salivary glands.** Contrast- enhanced T1 weighed magnetic resonance coronal imaging of the head showing massive thickening of the infraorbital nerve (red arrow). **B**: Histopathological examination of orbital muscle (upper panel) and parotid gland biopsies (lower panel) reveals dense lymphoplasmacellular infiltration and fibrosis (left pictures, HE staining) with dense infiltration of IgG4- producing plasma cells (right pictures, anti-IgG4, rabbit monoclonal, 1:1000 a.r., Abcam, Cambridge, UK).

When we saw the patient first in 2012, her medication included azathioprine 100 mg and prednisone 5 mg per day. She reported dry eyes and mouth, which had not changed during the last years. Otherwise, she was in good condition. Upon physical examination, mild swelling of the right submandibular gland was evident. On imaging, we found massive thickening of the ocular nerves (Figure 1A). Laboratory examination revealed negative anti-nuclear antibodies (ANA), extractable nuclear antigens (ENA), anti-neutrophil cytoplasmic antibodies (ANCA), rheumatoid factor, normal complement C3 and C4, and no cryoglobulins. Erythrocyte sedimentation rate (ESR), C- reactive protein (CRP) and blood chemistry were within normal range. However, total IgG levels were slightly elevated with IgG4 being strongly elevated (519 mg/dl, normal 5 – 125 mg/dl).

We retrieved biopsies of the ocular muscles, parotid gland and the left submandibular gland and asked the pathology department for re-evaluation of the specimens and staining for plasma cells, IgG and IgG4. All specimens revealed dense infiltration of IgG4- producing plasma cells with a highly increased tissue IgG4/IgG ratio of >40% (Figure 1B). Further, tissue eosinophilia, storiform fibrosis and obliterative phlebitis were present, thus confirming the diagnosis of IgG4- RD.

### History

The first descriptions of IgG4-RD organ manifestations were published in the late 19th century. In 1892, Johann von Mikulicz - a scholar of Theodor Billroth - first described a patient with an inflammatory disease of the salivary glands (Mikulicz’ syndrome) [[1]]. Subsequently, Küttner described in 1896 a patient suffering from a tumor-like lesion of the submandibular glands (Küttner’s tumor) [[2]]. In the same year, Riedel reported on “eisenharte Struma”, a condition characterized by a stone-hard, fibrous transformation of the thyroid gland and adjacent tissue [[3]]. However, the true nature of these disease manifestations remained unclear for another century.

Many organ manifestations of IgG4-RD including among others retroperitoneal fibrosis, sclerosing cholangitis, and sclerosing pancreatitis have since been described. Already decades ago, the systemic nature of the disease was recognized as evidenced by patients presenting with more than one organ involvement. For instance, Bartholomew et al. reported in 1963 on two patients with a combination of Riedel’s thyroiditis, retroperitoneal fibrosis and sclerosing cholangitis [[4]].

Significant progress was made in 1995, when a patient with a steroid- responsive form of pancreatitis was reported of presumed autoimmune origin (autoimmune pancreatitis, AIP) [[5]]. Later, in 2001 Hamano et al. reported elevated IgG4 levels in AIP patients and, finally, in 2002 infiltration of IgG4-bearing plasma cells in tissue samples from patients with “sclerosing pancreatitis” (as well as in concomitant retroperitoneal fibrosis) were documented by the same group [[6],[7]].

These findings have been replicated in different cohorts of patients with the above- described syndromes [[8],[9]]. Thus, a distinct disease entity comprising patients presenting with inflammatory and/or fibrosing single or multiple organ lesions in conjunction with elevated serum IgG4 levels and tissue IgG4 production became clear. While different names for this syndrome were used in the past 10 years, the term “IgG4- related disease” has recently evolved as most appropriate [[10]]. Consequently, the organ manifestations of IgG4-RD were renamed and eponyms were abandoned (Table 1).

**Table 1:** **Nomenclature for manifestations in different organ systems [adapted from Stone JH et al. A&R** [[10]]**]**

**Pancreas**	IgG4-related pancreatitis (Type 1 autoimmune pancreatitis)
**Bile ducts, gallbladder and liver**	IgG4-related sclerosing cholangitis
	IgG4-related cholecystitis
	IgG4-related hepatopathy
**Thyroid gland**	IgG4-related thyroid disease
**Salivary and lacrimal glands**	IgG4-related sialadenitis
	IgG4-related parotitis
	IgG4-related submandibular gland disease
	IgG4-related dacryoadenitis
**Orbits**	IgG4-related ophthalmic disease
	IgG4-related orbital inflammatory pseudotumor
	IgG4-related pan-orbital inflammation
	IgG4-related orbital myositis
**Retroperitoneal fibrosis, arteries**	IgG4-related retroperitoneal fibrosis
	IgG4-related aortitis/periaortitis
	IgG4-related periarteritis
**Intrapulmonary, mediastinal and pleural involvement**	IgG4-related lung disease
	IgG4-related mediastinitis
	IgG4-related pleuritis
**Lymph nodes**	IgG4-related lymphadenopathy
**Kidney**	IgG4-related kidney disease
	tubulointerstitial nephritis secondary to IgG4-related disease
	membranous glomerulonephritis secondary to IgG4-related disease
	IgG4-related renal pyelitis
**Miscellaneous**	IgG4-related perineural disease*
	IgG4-related pachymeningitis
	IgG4-related hypophysitis
	IgG4-related mesenteritis
	IgG4-related mastitis
	IgG4-related prostatitis
	IgG4-related epididymo-orchitis*
	IgG4-related paratesticular pseudotumor*
	IgG4-related skin disease
	IgG4-related pericarditis

### Diagnosis and pathology

IgG4-RD can affect any organ or tissue. Thus, the clinical picture is highly heterogeneous. While some patients may present with a single site involved, others may have a few or many organs affected by IgG4-RD. These may arise synchronously or metachronously. The disease may manifest as a tumefactive lesion or a rather diffuse infiltrative process, further contributing to a diverse clinical picture. In addition, some organs show a distinct involvement, as for instance lymphadenopathy, periaortitis, or a secondary form of membranous glomerulonephritis (GN) [[11]–[13]].

Laboratory findings in IgG4-RD are often inconspicuous. Inflammatory markers such as ESR and CRP may be highly elevated, but can be normal despite active disease in a substantial proportion of patients. Anti-nuclear antibodies, anti- SS-A as well as anti- SS-B antibodies are negative in the majority of patients, while low complement levels (C3 and C4) are not uncommon [[14],[15]]. Polyclonal hypergammaglobulinemia is often found in IgG4-RD. Increased serum IgE levels and allergic diseases are present in about one third of patients [[16]]. IgG subclass analyses reveal highly elevated serum IgG4 levels in many but not all patients. It should be underlined that IgG4 levels can be substantially misleading when they are used as a sole criterion for diagnosis (or exclusion) of IgG4-RD. On the one hand, a number of other diseases, such as cancer, infections and autoimmune diseases, including vasculitis, are associated with increased IgG4 levels [[17]]. On the other hand, a number of IgG4-RD patients may have normal IgG4 levels. Thus, the sensitivity of IgG4 in IgG4-RD was found to be 90% and the specificity 60% in one study [[18]]. In another study the positive predictive value of an elevated serum IgG4 for IgG4-RD was found to be as poor as 10% [[19]].

In some patients with active IgG4-RD IgG4 levels were found to be inappropriately low due to the prozone effect, a situation where too many antibodies prevent agglutination of antigenic particles in the test system. If disease activity is high, but IgG4 levels are low, sample dilution may lead to correction of the prozone effect and reveal – appropriately – much higher IgG4 levels in particular patients [[20]].

**Figure 2:**
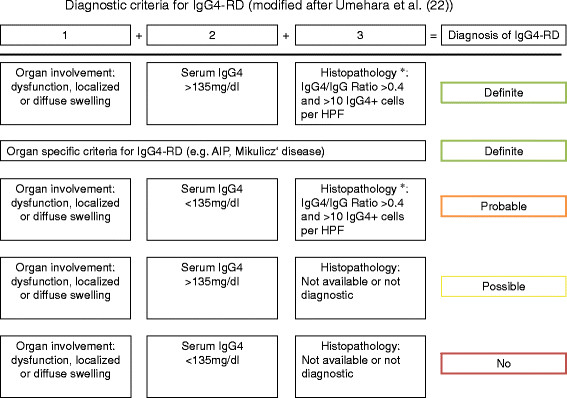
**Comprehensive diagnostic criteria for IgG4- related disease (modified after Umehara et al.** [[22]]**).** *Histopathology suggestive of IgG4-RD, e.g. lymphoplasmacytic infiltration and fibrosis.

While the location of disease manifestation may vary, the histological pattern is – with some exceptions – more or less uniform and shares particular features: (i) storiform fibrosis (resembling the spokes of a cartwheel), (ii) a dense lymphoplasmatic infiltrate with an increased number of IgG4+ plasma cells (at least > 10/high power field (HPF) – depending on the particular organ) and/or an increased IgG4/IgG ratio (usually >40%) and (iii) an obliterative phlebitis. Tissue eosinophilia is also very characteristic. The presence of neutrophils, granulomas, neutrophilic microabscesses, and necrotizing vasculitis strongly argues against IgG4-RD [[21]]. In some organs, IgG4-RD can manifest with distinct histopathological features, e.g. in kidney and lymph nodes (see section on clinical manifestations). Recently, a Japanese study group proposed diagnostic criteria for IgG4-RD in general (Figure 2) [[22]]. In addition, there have been proposals for diagnostic criteria of organ- specific IgG4-RD manifestations such as in kidney or pancreatic disease [[23],[24]].

### Epidemiology

There are few studies reporting on the epidemiology of IgG4-RD, mainly derived from Asian studies. A cross-sectional study in 2009 estimated that approximately 8000 individuals in Japan were suffering from IgG4-RD, thus accounting for a prevalence of approx. 60 affected individuals per million inhabitants [[25]]. Slightly more males were affected and mean age was 58 years well in line with previous reports. There are no data on the prevalence of IgG4-RD in Europe or elsewhere, but IgG4-RD is thought to be rarer in these areas. IgG4-RD usually affects middle-aged individuals and at least for some organ manifestations such as type 1 AIP a male predominance is evident.

### Pathophysiology

Little is known about the initiation process of IgG4-RD or how and why these specific organ infiltrations occur. There is some evidence from Asian populations, that certain HLA alleles – particularly DRB1*0405 and DQB*0401 – increase the susceptibility to type 1 AIP [[26]]. Further, few single- nucleotide polymorphisms (SNP) in genes related to immune system such as CTLA-4 and Fc- receptor-like 3 have been linked to IgG4-RD [[27],[28]]. In AIP, autoantibodies against the plasminogen- binding protein of H. pylori were found in the majority of patients in one study [[29]]. Plasminogen- binding protein of H. pylori shares homology to a protein expressed in pancreatic acinar cells. The authors hypothesized, that through molecular mimicry this immune response could lead to IgG4-RD.

It is suspected but unclear, whether IgG4-RD truly belongs to the group of autoimmune disorders. There is evidence of autoantibodies such as ANA, rheumatoid factor, and others in some patients [[30]]. However, these autoantibodies are far beyond from being specific for IgG4-RD. The same is true for organ- specific autoantibodies to proteins expressed for instance in the pancreas or bile ducts [[31]].

In contrast to many autoimmune disorders, IgG4-RD seems to have a skewed T- cell response towards a TH2 phenotype. Increased tissue levels of TH2-cytokines such as Interleukin-4 (IL-4), −5 (IL-5) and −13 (IL-13) were found in IgG4-RD [[32]]. Further, 40% of IgG4-RD patients have increased serum IgE levels and allergic diseases are common [[16]]. Moreover, tissue eosinophilia is typical for IgG4-RD [[33]]. Some studies also suggested an increased activation of regulatory T cells (Treg), which might be due to the over-expression of transforming growth factor β (TGF-β), an important regulator of Treg development [[34],[35]]. Previously it could be demonstrated that the expression of IgG4 class switch-related molecules is different in labial salivary glands and peripheral blood mononuclear cells. In glands of IgG4-RD patients upregulated Treg cytokines such as IL-10 and TGF-β appear to play pathogenic roles in IgG4-specific class-switch recombination and fibrosis. Activation-induced cytidine deaminase (AID), as a marker of nonspecific immunoglobulin class-switch recombination, was also found to be increased in labial salivary glands of IgG4-RD patients and could contribute to upregulation of IgG4-specific class-switch recombination. IgG4 class-switch recombination seemed to be mainly upregulated in affected organs [[36]].

Well-fitting to a TH2 immune response, IgG4-RD is characterized by both systemic and localized IgG4 production. It is currently unclear, whether these antibodies are acting as pathogenic antibodies or are just a bystander phenomenon. In healthy individuals, IgG4 is the least abundant IgG subclass molecule accounting for less than 5% of total IgG [[37]]. In addition, IgG4 itself seems to be unable to activate the classic complement pathway. Another characteristic feature of IgG4 antibodies is the possible occurrence of Fab arm exchange of these antibodies [[38]]. This is due to weak bonds between the heavy chain of the IgG4 antibodies thus allowing the Fab arm exchange and formation asymmetric bispecific antibodies. This further hampers antigen cross-linking and immune complex formation of IgG4 antibodies. Nonetheless, there is evidence of importance and pathogenicity of IgG4 antibodies in human diseases. Certain autoantibody immune mediated diseases such as membranous GN and thrombotic thrombocytopenic purpura seem to be mediated by antibodies of the IgG4 type [[39],[40]].

Interestingly, low C3 and C4 complement levels can be observed in one third of IgG4-RD patients with renal involvement indicating immune complex formation [[15]]. In type 1 AIP, tissue deposition of C3c, IgG4 and IgG can be observed at the basement membrane of pancreatic and bile ducts and of acini [[41]]. C3 and IgG deposition can also be observed at the tubular basement membrane in IgG4- related tubulointerstitial nephritis (TIN) [[15]]. Thus, immune complex mediated tissue damage may occur at least in some organ manifestations of IgG4-RD.

Recent insights into the pathophysiology of IgG4-RD suggest that one of the major drivers of the disease appear to be plasmablasts. Oligoclonal expanded plasmablasts with extensive somatic hypermutation were found to be increased in IgG4-RD patients with active disease. Of interest, depletion of B cells with rituximab leads to a reduction of these plasmablasts and correlates with disease remission [[42]]. Compared to other inflammatory diseases before treatment and healthy controls, circulating plasmablasts are elevated in patients with active IgG4-RD, even in those patients with normal serum IgG4 concentrations. This observation poses plasmablasts as potential biomarkers for diagnosis and assessment of treatment response [[43]].

### Clinical manifestations

#### Pancreas

Type 1 AIP is the classical presentation of IgG4-RD, while type 2 AIP is not part of the IgG4 spectrum, has distinct histopathological features such as ductal neutrophilic abscesses and ductal injury and is not reviewed here in detail [[44]]. Type 1 AIP is rare, being the cause of chronic pancreatitis in less than 5% of cases. It typically occurs in middle-aged males [[45]]. IgG4 serum levels are usually elevated, but are normal in up to 20% of patients. In contrast, elevated levels of IgG4 are uncommon in type 2 AIP [[46]]. Obstructive jaundice is a common symptom at presentation, while pain may be absent [[44],[45]]. In addition, extra-pancreatic organ involvement is frequent in type 1 AIP, thereby representing the systemic nature of IgG4-RD.

Imaging techniques can provide clues to the diagnosis of AIP. Magnetic resonance cholangiopancreatography (MRCP) and endoscopic retrograde cholangiopancreatography (ERCP) may demonstrate pancreatic duct strictures and/or stricture of the common bile duct as well as irregular narrowing of intrahepatic ducts. Complete obliteration of the bile duct is unusual. Computed tomography (CT) and magnetic resonance (MR) imaging show focal or diffuse pancreatic enlargement, a peripancreatic capsule-like rim, and late phase contrast-enhancement, which is believed to correspond to peripheral inflammation and fibrosis [[38],[47]].

While radiological and serological studies as well as clinical symptoms may help to establish the diagnosis, in less characteristic cases the final diagnosis needs to be based on histological tissue examination. In type 1 AIP a typical diffuse lymphoplasmacytic infiltrate and a storiform fibrosis are evident. IgG4-positive plasma cells are usually found >50 per high-power field (HPF). Obliterative phlebitis is common, but may be masked by the inflammatory infiltrate. In such cases, an elastic stain can be helpful. Tissue eosinophils are also frequently found. Of note, while there may be a periductal infiltrate, the ductal epithelium – in contrast to type 2 AIP - is preserved [[48]]. Diagnostic criteria for the diagnosis of AIP have been published [[24]].

Unfortunately, AIP shares many similarities with pancreatic cancer, such as age, male predominance, painless jaundice or swelling of the pancreas [[49]]. This may be of special relevance in the case of “localized” AIP, which can lead to a tumorous appearance of the pancreatic head. In one large series 2.2% of patients undergoing pancreaticoduodenectomy were diagnosed retrospectively with AIP (“lymphoplasmacytic sclerosing pancreatitis”). Of these cases the preoperative diagnoses were pancreatic cancer in 53%, periampullary neoplasm in 38%, and cholangiocarcinoma in 9% [[50]]. Thus, a thorough work up is essential before either surgery or steroid treatment is planned. Of note, solely relying on elevated IgG4 levels may be misleading, as these may also be increased in pancreatic cancer. The notion that pancreatic cancer can develop in patients with definitive AIP further complicates the management of these patients [[51]].

#### Bile ducts, gallbladder and liver

The most characteristic clinical sign of IgG4- related cholangitis at presentation is obstructive jaundice. IgG4-related cholangitis typically affects large bile ducts, while small duct lesions are variable [[52],[53]]. In addition to the bile ducts, the gallbladder seems to be involved in a substantial proportion of cases [[54]]. The intrapancreatic bile duct as well as more proximal parts of the ducts are typically affected. A localized diffuse thickening of the bile duct is characteristic, but multiple strictures may occur. Increased IgG4 levels are found in approximately 80% of the patients. Strikingly, 92% of patients in both mentioned series had concomitant type 1 AIP.

Histological features of IgG4-related cholangitis are similar to those found in other organs affected by IgG4-RD. Liver biopsies may be inconclusive for a final diagnosis of IgG4-related cholangitis. However, a high number of patients with AIP demonstrate pathological features on liver biopsies. The picture does not appear to be uniform in all patients and is composed of portal inflammation (with or without interface hepatitis), large bile-duct obstructive features, portal sclerosis, lobular hepatitis, and canalicular cholestasis. Multiple features can coexist in single patients [[55]]. Whether there exists a true “IgG4-related autoimmune hepatitis” or not is a matter of discussion, but a minority of patients with autoimmune hepatitis may in fact have an IgG4-RD [[56]].

If patients are diagnosed with type 1 AIP and have in addition characteristic manifestations on imaging in combination with elevated IgG4 levels, then a diagnosis of IgG4 related cholangitis is usually straightforward. However, in the absence of AIP a histological tissue examination will be required in most patients. Different strategies for tissue sampling are possible. Ampullary biopsies are rather easy to obtain. Sensitivity for IgG4 positive cells is 50%, but samples from this site are lacking information on storiform fibrosis. Bile duct biopsies, which are more challenging to perform, have the same sensitivity for IgG4 positive cells but can demonstrate the full spectrum of pathological features of IgG4-RD. Liver biopsies are invasive and have a sensitivity of only 20% for IgG4 positive cells. However, liver biopsy may be especially helpful in the consideration of differential diagnoses [[53]].

Neoplastic disease is a main differential diagnosis of IgG4-RD in bile ducts, gallbladder, and liver. Cholangiocellular carcinoma has to be excluded and the differentiation of carcinomas to IgG4-RD can provide difficulties [[57]]. IgG4 levels can be misleading, as patients with cholangiocarcinoma – as well as with other forms of cancer and a broad range of other diseases – can have moderately elevated IgG4 levels [[57]]. Further potential mimics of IgG4-related sclerosing cholangitis include follicular cholangitis, primary sclerosing cholangitis and sclerosing cholangitis with granulocytic epithelial lesion (the latter probably being the bile duct counterpart of type 2 AIP) [[58]–[60]].

#### Thyroid gland

Since 2010, Riedel’s thyroiditis is considered part of the IgG4-RD spectrum [[61]]. In contrast to most manifestations of IgG4-RD, Riedel’s thyroiditis has a female preponderance. Riedel’s thyroiditis may affect a lobe or the total thyroid gland. A specific finding is the extension of the fibrotic mass into adjacent tissues. This process can lead to invasion of the parathyroid glands (thereby causing hypoparathyroidism), skeletal muscles, nerves, blood vessels, as well as the trachea [[62]]. Clinical symptoms include pain, local swelling, dysphagia, hoarseness, or symptoms due to tracheal narrowing [[63],[64]]. A definitive diagnosis requires histopathologic examination. Indeed, most patients undergo surgery because of suspected malignancy, as fine needle aspiration is frequently inconclusive. At surgery a clear differentiation between anatomic structures is often difficult due to the invasive nature of the fibrotic mass. Therefore, a complete thyroidectomy is seldom performed. Recently, the fibrosing variant of Hashimoto’s thyreoiditis, which remains localized to the thyroid gland, emerged as being also a part of the IgG4-RD spectrum [[65]].

#### Salivary and lacrimal glands

Salivary and lacrimal glands are frequently affected in IgG4-RD. Chief complaints include a persistent asymmetric or symmetrical swelling of the involved glands and impaired secretion. Pain is uncommon [[66]]. IgG4-related sialadenitis and dacryoadenitis seem to affect both sexes equally. Age at onset shows a wide range with a mean of approximately 60 years [[67],[68]]. Chronic sclerosing sialadenitis can present with mass lesions ranging up to 5 cm. Microscopic examination shows some distortion but overall preservation of the lobular architecture and a dense lymphoplasmacytic infiltrate with numerous hyperplastic lymphoid follicles. The classical features of IgG4-RD are usually found. A similar picture is found in IgG4-related dacryoadenitis [[69],[70]].

One of the major mimics of Mikulicz disease is SS [[71]]. In fact, formerly Mikulicz syndrome and SS were considered closely related [[72]]. From a clinical perspective, SS usually never affects the submandibular glands isolated. In contrast to IgG4-RD, SS patients frequently have anti-SS-A and/or anti-SS-B autoantibodies but do not generally exhibit elevated serum IgG4 levels [[66]]. It is very likely that a substantial proportion of patients with “ANA negative SS” will in fact have IgG4-RD. Histopathological differences are helpful to distinguish both diseases. IgG4+ plasma cell infiltration in tissue is frequent in patients with IgG4-RD, but not in SS. Expansion of IgG4+ plasma cells with fibrosis or sclerosis is an important histopathological finding in IgG4-RD, but unusual for SS. Lymphocytic follicle formation is commonly observed in IgG4-RD, but lymphocytic infiltration in the ducts (formation of lymphoepithelial lesions) is rare [[73]].

Other diseases that need to be distinguished from IgG4-related sialadenitis and dacryoadenitis are lymphomas, sialolithiasis and carcinomas. The latter may be surrounded by an inflammatory infiltrate containing IgG4 positive plasma cells complicating the exact diagnosis [[69]].

#### Orbits

Orbital tumors of the ocular adnexa consist of a heterogeneous group. A large proportion of these tumors are lymphoproliferative disorders (LPDs). These LPDs represent malignant lymphomas and a diverse group of orbital inflammations, such as reactive lymphoid hyperplasia or infiltration [[74]]. Several structures within the orbital cavity and adjacent structures such as extraocular muscles, nerves (including the supra- and inraorbital as well as optic nerve), periorbital membrane and lacrimal sac can be affected. Chronic lid swelling and proptosis are common clinical signs. Pain is unusual. If extraocular muscles are involved eye motility can be restricted. Visual disturbances can occur if the optic nerve is involved [[75]]. Lesions may appear as a tumefactive mass [[76],[77]]. The fibroinflammatory tissue can extend into the pterygopalatine fossa and cavernous sinus. Bone destruction is an unusual sign, but has been reported [[78],[79]].

Pathohistologically, characteristic signs of IgG4-RD can be found, but obliterative phlebitis seems to be rare [[75]]. Rather, fibrosis is a prominent feature, which may relate also to duration of disease. However, the storiform pattern seems to be less common than in other organs affected by IgG4-RD [[69]].

#### Retroperitoneal fibrosis and large vessel involvement

Retroperitoneal fibrosis (RPF) is a rare disease characterized by the occurrence of fibrosis in the retroperitoneum. RPF usually affects adult middle-aged males and is strongly associated with smoking [[80]]. Clinical manifestations can vary considerably. In some patients findings may be incidental, other patients present with back pain. In some cases manifestations of IgG4-RD in other organs leads to work up, thereby elucidating retroperitoneal fibrosis and/or aortitis. Hydronephrosis due to ureteral entrapment is a common complication [[81]]. Periaortitis is asymptomatic in most patients. In rare cases a rupture after aneurysmal transformation can occur [[82]].

On CT imaging circumferential arterial wall thickening, which is caused by sclerosing inflammation in the adventitia, is characteristic. Plaque-like lesions can also be seen. Of note, perivascular lesions are usually prominent. Affected vessels demonstrate homogeneous enhancement at the late phase of contrast–enhanced CT. Some cases show dilated lumen of the affected vessels. Arteries penetrating the fibroinflammatory process surrounding the aorta are usually patent [[81]]. Histopathology can show typical features of IgG4-RD, but in long-standing disease, fibrosis rather than inflammation may be the dominant finding.

RPF is closely linked to inflammatory aortic aneurysm and thoracic aortitis and is therefore now being summarized as chronic periaortitis [[83]]. RPF itself seems to be a heterogeneous group of diseases. Some patients show signs of IgG4-RD but many patients have isolated RPF [[84]]. Inflammatory abdominal aortic aneurysms may also be part of the IgG4-RD spectrum [[85]]. IgG4-RD mainly affects the aorta but also frequently involves its main branches. In one study 9% of noninfectious aortitis cases of the thoracic aorta were caused by IgG4-RD [[86]]. Of interest, while both the thoracic aorta and the infrarenal aorta are predominant sites of involvement the suprarenal abdominal aorta seems to be spared [[81]]. Besides the aorta and its main branches, also peripheral arteries are sites of involvement of IgG4-RD [[87]]. IgG4-related aortitis mainly involves the adventitia. However, the media can also be affected and the lamellar elastic fibers can be disrupted. The latter seems to be associated with aneurysmal transformation [[12],[81]].

#### Intrapulmonary, mediastinal and pleural involvement

IgG4-RD can affect the airways, pleura and mediastinum. Patients may present with cough, dyspnea, chest pain or frequently remain asymptomatic [[88],[89]]. Intrapulmonary manifestations include nodules and mass lesions (“inflammatory pseudotumors”) but may also present as interstitial lung disease. In the visceral and parietal pleura, IgG4-RD can cause nodular lesions [[90]]. In the same patients, bronchial inflammation, edema, and stenosis may occur [[91]]. In addition, mediastinal fibrosis is another clinical syndrome proposed to lie within the spectrum of IgG4-RD.

Imaging studies reflect the diverse nature of morphological manifestation of IgG4-related lung disease. A recent study categorized lung findings on imaging into four major subtypes: solid nodular type (nodule or mass); (multiple) round-shaped ground-glass opacity type; alveolar interstitial type (bronchiectasis, honeycombing and diffuse ground-glass opacities); and bronchovascular type (with thickening of bronchovascular bundles and interlobular septa) [[89]].

Diagnosis of IgG4-RD in the lungs can be challenging. The lung tends to show a stereotypic morphologic response to a variety of different injuries. Thus, differential diagnostic considerations can be difficult. While fibrosis and obliterative vascular changes are common in solid areas, a characteristic storiform pattern may be lacking on tissue samples. While eosinophils are common as it is the case in other sites of IgG4-RD manifestations, neutrophils may also be seen [[88],[92]]. A characteristic feature of IgG4-related lung disease is that pulmonary veins as well as arteries can be affected [[90]]. A number of diseases can mimic IgG4-related lung disease, including lung cancer, idiopathic interstitial pneumonia, LPD, and sarcoidosis [[89]]. Thus, the field of differential diagnoses is broad.

#### Lymph nodes

Localized or systemic involvement of lymph nodes is common in IgG4-RD [[93]]. Differential diagnosis is broad including lymphomas, metastatic disease, Castleman’s disease and other immune mediated or hematological diseases. Fever, weight loss and night sweats, however, are uncommon in IgG4-RD [[94]]. Interestingly, histopathology of lymph nodes in IgG4-RD differs from the usual findings. Storiform fibrosis and obliterative phlebitis are usually absent in affected lymph nodes [[11]]. Findings include the following: multicentric Castleman’s disease-like, reactive follicular hyperplasia-like, interfollicular expansion and immunoblastosis; progressively transformed germinal center type; and inflammatory pseudotumor-like IgG4-related lymphadenopathy [[94]]. It is important to recognize that the histopathology of lymph nodes in suspected IgG4-RD is usually not straightforward when used as a sole criterion for diagnosis. Thus, it may be difficult to clearly distinguish IgG4-related lymphadenopathy from other disease. However, it may be important to take lymph node biopsies to exclude e.g. malignancy.

#### Kidney

The main manifestations of IgG4-RD in the kidney is tubulointerstitial nephritis and membranous GN [[95],[96]]. The disease can clinically present as acute or chronic renal failure, renal mass lesions, or both. Contrast-enhanced CT scans of patients with IgG4-related tubulointerstitial nephritis shows mostly bilateral and multiple renal parenchymal lesions, predominantly involving the renal cortex. These lesions may appear as small peripheral cortical nodules, well defined or ill-defined round lesions, wedge-shaped lesions or diffuse patchy. In some cases a mass effect can be present. Extraparenchymal involvement can be found in the form of a diffuse rim of soft tissue surrounding the kidney, irregular nodules in the renal sinuses or diffuse wall thickening of the renal pelvis [[97]].

Histopathology shows typical fibrosis and a lymphoplasmacytic infiltrate with a high number of IgG4 positive cells. IgG and/or C3 granular deposits on the tubular basement membrane are present in a significant number of patients. In addition, a variety of glomerular lesions, mainly different forms of glomerulonephritis, may be additionally present in such patients. All this has raised the suspicion that immune complexes are involved in the pathogenesis of IgG4-related tubulointerstitial nephritis [[15]]. Of interest, in some cases tubules are destroyed [[98]]. This seems to be somewhat in contrast to other manifestations of IgG4-RD, where the epithelium is usually preserved (e.g., salivary glands, bile ducts and pancreas). In some patients, a secondary form of membranous GN develops lacking classical IgG4-RD histopathological features such as storiform fibrosis or obliterative phlebitis. Of interest, a substantial proportion of patients with idiopathic membranous nephropathy have autoantibodies of the IgG4 class that bind to the M-type phospholipase A2 receptor. In contrast, patients with IgG4RD do not show such antibodies, regardless of the presence or absence of membranous nephropathy [[13],[99]].

#### Miscellaneous

Several structures of the nervous system were found to be involved in IgG4-RD. Peripheral nerves have been reported to be affected by IgG4-RD. Common sites of manifestations are the orbital and spinal nerves with predominant infiltration of the epineurium (“perineural disease”). Of interest, neurologic symptoms are seldom which seems to be related to the fact that nerve fascicles are usually intact and not damaged [[100]]. In the pituary gland, IgG4-RD disease can cause hypophysitis [[101],[102]]. IgG4-related inflammatory pseudotumors may arise directly within the central nervous system [[103]]. Roughly one third of cases with hypertrophic pachymeningitis appear to be due to IgG4-RD [[104]].

Idiopathic cervical fibrosis, a rare sclerosing disease of the neck, seems to be part of the IgG4-RD spectrum [[105]]. A unusual form of mastitis, sclerosing mastitis, which can present as painless masses in the breasts, has also been reported to be a manifestation of IgG4-RD [[106]]. In men, IgG4-RD can be the cause of prostatitis, orchitis as well as paratesticular pseudotumors [[107]–[109]]. In some cases IgG4-RD can involve the skin, presenting as erythematous and itchy plaques or subcutaneous nodules [[110]]. The pericardium has been reported to be a site of manifestation of IgG4-RD, resulting in constrictive pericarditis [[111],[112]]. Eosinophilic angiocentric fibrosis, a rare tumefactive lesion of the orbit and upper respiratory tract, has also been supposed to be a manifestation of IgG4-RD [[113]].

### Treatment

Overall, there is no evidence for treatment options from randomized controlled trials. Clinical experience is that most IgG4-RD patients respond favorably to glucocorticoid (GC) treatment. The effect of GCs has been reported for several sites of IgG4-RD manifestations in different cohort studies. While it appears that spontaneous remissions do occur [[114]], the use of GCs induces remission in most patients and earlier than without treatment. However, relapse is frequently seen after GCs are tapered or stopped [[52]]. Relapses may be confined to the originally affected organs but also occur in other organs. They may be preceded or accompanied by an increase in IgG4 levels [[115],[116]].

Whether treatment in patients with IgG4-RD needs to be established depends on the localization and extent of disease manifestation. In some patients with minor involvement (e.g. asymptomatic and incidentally found IgG4-related lymphadenopathy or small, indolent nodular lesions in IgG4-related lung disease), immediate treatment may not be indicated and follow-up may be more appropriate. In other cases with organ dysfunction or pseudotumors (e.g., renal involvement, AIP with bile duct obstruction and jaundice, lacrimal gland swelling, pachymenigitis, etc.) rapid introduction of therapy is necessary to avoid loss of organ function. Thus, treatment decision has to be individualized [[117]]. Most cohort studies report on IgG4-RD patients with a particular organ involvement, which may not translate to other organ manifestations. Moreover, a substantial proportion of publications are from Asian IgG4-RD patients, questioning whether these data also apply to non-Asian patients.

In general, most experts would start with a dosage of 40 mg prednisolone or 0.6 mg/kg of body weight per day and taper the dose over several months, starting with a first dose reduction after 2–4 weeks [[117]–[119]]. Treatment response is usually seen within two to four weeks. Major drawbacks of GCs are side effects as well as the need for maintenance therapy. Thus, a number of drugs such as azathioprine, mycophenolate mofetil or methotrexate have been used as GC sparing agent [[119]–[123]]. In addition, more intensive therapies such as cyclophosphamide, fludarabine and bortezomib have been reported to be of benefit in IgG4-RD patients [[123]–[125]].

A promising treatment strategy in GC- dependent or refractory patients is rituximab (RTX) [[84],[120],[126]]. RTX has been used in a case series of 10 refractory IgG4-RD patients. The duration of treatment effect is not established. Not surprisingly, relapse of IgG4-RD after rituximab treatment has been reported [[127]]. One major interesting finding was that RTX specifically reduced IgG4 levels, while the other IgG subclasses remained stable. This has led to the hypothesis that short lived plasma cells producing IgG4 are involved in the disease [[84],[120]].

## Conclusions and future perspectives

Over the last decade a number of previously thought unrelated diseases have been recognized as a spectrum of a single disease. Terminology and diagnostic criteria have since been worked out [[10],[22]]. However, as with every “new” disease a lot of enthusiasm might arise. This can lead to the situation that uncommon conditions are not only underdiagnosed, but eventually also overdiagnosed. While it is straightforward for most IgG4-RD manifestations to be accepted as part of the IgG4-RD spectrum, for other manifestations no final conclusions can be drawn to date, as only few cases have been reported, so far. Within the future it has to be clarified whether all of the nowadays suspected diseases truly belong to IgG4-RD spectrum.

Currently, GCs are considered the first line therapy, but randomized trials to determine optimal dose and duration are warranted. In addition, data on the effect of other immunosuppressants are very limited. Further points that need to be addressed in the future are whether all organ manifestations respond similar to treatment and, finally, it needs to be investigated whether non-Asian populations do behave similar to Asian patients, for whom most data are published to date.

Further, it remains unclear, how IgG4-RD patients should be followed. IgG4 seems to have some characteristics of a biomarker in patients with elevated IgG4 levels, but it is unclear whether IgG4 levels can be used to guide therapy for instance [[128]]. Future research will include the search for further biomarkers for diagnosis and/or follow up of IgG4-RD patients. One possible useful biomarker seem to be circulating plasmablasts [[42],[43]]. Whether expensive imaging modalities such as PET-CT, will become part of routine care of IgG4-RD patients or not, needs to be further investigated [[129]]. Finally, we do not understand the pathophysiology of the disease, which could lead to the invention of specific therapies for this rare disease.

## Competing interests

JZ received an unrestricted research grant for clinical research on IgG4-RD from Roche. JZ received honoraria for educational activities and advisory board participation from Roche.

## Authors’ contributions

HP and JZ conceived and designed the manuscript. IP, AW, BO and JZ collected clinical and pathological data of the case vignette. All authors participated in the literature review process and drafted the final manuscript. All authors and the Vasculitis and Orphan Diseases Working Group of the Austrian Society of Rheumatology read the final manuscript and gave approval for the version to be published.
